# The fetal-maternal interface of the nine-banded armadillo: endothelial cells of maternal sinus are partially replaced by trophoblast

**DOI:** 10.1186/s40851-016-0048-1

**Published:** 2016-06-08

**Authors:** Arun Rajendra Chavan, Günter P. Wagner

**Affiliations:** Department of Ecology and Evolutionary Biology, Yale University, New Haven, CT USA; Yale Systems Biology Institute, Yale University, West Haven, CT USA; Department of Obstetrics, Gynecology and Reproductive Sciences, Yale Medical School, New Haven, CT USA; Department of Obstetrics and Gynecology, Wayne State University, Detroit, MI USA

**Keywords:** Fetal-maternal interface, Fetal-maternal tolerance, *Dasypus novemcinctus*, Nine-banded armadillo, Placental fibrinoid

## Abstract

**Background:**

The evolution of invasive placentation in the stem lineage of eutherian mammals entailed resolution of the incompatibility between a semi-allogenic fetus and the maternal immune system. The haemochorial placenta of nine-banded armadillo (*Dasypus novemcinctus*) is thought to conceal itself from the maternal immune system to some degree by developing inside a preformed blood sinus, with minimal contact with the uterine connective tissue. In the present study, we elucidate the micro-anatomical relationship between fetal and maternal tissue of the nine-banded armadillo using histochemical and immunohistochemical tools.

**Results:**

We conclude that the chorio-allantoic villi are separated from the myometrium by a vascular endothelial layer, as previously proposed. However, we also observe that the trophoblast cells establish direct contact with the endometrial stroma on the luminal side of the endometrium by partially replacing the endothelial lining of the sinus. Further, we demonstrate the presence of leukocytes, perhaps entrapped, in the placental fibrinoids at the interface between the intervillous space and the endometrial arcade.

**Conclusions:**

The trophoblast of the armadillo invades the uterine tissue to a greater extent than was previously believed. We discuss the implications of this finding for the fetal-maternal immune tolerance.

## Background

Viviparous mammals exhibit one of three types of placentation; in order of increasing invasiveness of the placenta, these are epitheliochorial, endotheliochorial, or haemochorial [1]. Epitheliochorial placentation (e.g., in most marsupials, cattle, sheep) is non-invasive because the chorion does not establish sustained contact with uterine stroma. The other two types of placentation are invasive, meaning that the fetal chorion is in a sustained (>2 days) interaction with the endometrial stoma and/or maternal blood. Chorion invades only the luminal epithelium and contacts the maternal vascular endothelium in an endotheliochorial placenta (e.g., in carnivores, elephants), while it erodes both the luminal epithelium and the endothelium to directly contact the maternal blood in a haemochorial placenta (e.g., in human, rodents a. o. m.). Both types of invasive placentation lead to a direct and extended apposition of the fetal tissue and endometrial connective tissue.

Invasive placentation evolved in the stem lineage of eutherian mammals (also known as placental mammals) [2–4]. Invasive placentation faces two physiological challenges: (i) destruction of maternal tissue by the blastocyst induces an inflammatory reaction, and (ii) the invading blastocyst is semi-allogenic relative to the mother. That is, about half of the genes expressed by the fetus are of paternal origin, and thus are potential alloantigens for the maternal immune system [5]. Evolution of invasive placentation in eutherian stem lineage necessitated the evolution of mechanisms to control inflammation [6], and to allow for the intrauterine growth of the fetus without undergoing rejection by the maternal immune system. Immunology at the fetal-maternal interface is an area of active research, and numerous mechanisms of immune tolerance have been discovered in mouse and human (reviewed in [7]).

Here we investigate the fetal-maternal interface of the nine-banded armadillo (*Dasypus novemcinctus*). Armadillo belongs to the eutherian clade Xenarthra. Xenarthra and the well-studied Euarchontoglires (e.g., mouse and human) bracket the entire diversity of eutherian mammals [8–10]. Its phylogenetic placement makes Xenarthra a critical lineage for better understanding the evolutionary origin of eutherian pregnancy.

Armadillo has a haemochorial placenta, but the manner in which the haemochorial arrangement is achieved is considerably different from that of other species with haemochorial placentation [11–14], as described below.

Enders [12] has divided armadillo placental development into three phases: (i) avillous phase of initial attachment, (ii) establishment and expansion of villous placenta, and (iii) phase of mature (fully developed or definitive) placenta. The initial site of attachment of the blastocyst is the most distal (i.e., anterior) tip of the fundus, at the intersection of two prominent mucosal folds perpendicular to each other [15]. Endometrium contains preformed blood sinuses, even in the non-pregnant stage and during diapause, which are close to the luminal surface in the fundus [16]. The blastocyst attaches to the fundic mucosa by the trophoblast layer on the embryonic pole (also known as the Rauber’s layer). Subsequently, the Rauber’s layer forms an annular thickening at the periphery of the initial site of attachment. Trophoblast on the abembryonic pole degenerates in the meantime, consequently giving rise to an inverted yolk-sac placenta towards the uterine corpus, in addition to the chorio-allantoic placenta being formed on the embryonic side.

In the second phase, luminal epithelium is penetrated by the trophoblast, and villous placenta is established in the preformed blood sinuses. Trophoblast invades luminal epithelium at limited areas, and sends fingerlike projections into the preformed blood sinuses that lie closely underneath the epithelium. These trophoblastic projections mature into chorio-allantoic villi upon development of mesodermal cores from the allantois. Further development and branching of the villi takes place within the blood sinuses, and is accommodated by expansion of the sinuses, rather than by destruction of maternal tissue. Maturation of the placenta is complete when fetuses are 6–7 cm in length, and is marked by the disappearance of the cytotrophoblast, leaving the villi without any cytotrophoblastic cell columns at their distal tips. From the disappearance of cytotrophoblast to parturition is the phase of mature or definitive placenta in which no more villous growth is thought to take place.

In the mature placenta, the myometrium and the blood sinuses are not separated by much endometrial stromal tissue, and as a consequence, armadillo lacks a *decidua basalis* between the placental villi and myometrium. Endometrial tissue is retained in small islands on the myometrial side, and on the luminal side of the villi in the form of a thin arch over the villous tree. This remnant of endometrium is not a *decidua capsularis*, as it encapsulates only the placenta, not the fetus. Enders and colleagues [16] aptly refer to it as the *endometrial arcade*. The maternal blood sinus can be considered as the intervillous space of the placenta since villi develop and lie within the sinus. Note that the haemochorial configuration is reached without extensive damage to maternal tissue except at the original sites of penetration.

In this study we describe the microanatomy of the interface between the placental villi and uterus. We show that the long-held idea that the armadillo trophoblast makes no physical contact with the endometrial connective tissue, by growing within a blood sinus lined by an uninterrupted vascular endothelium [12, 17], is valid for the myometrial side, but not for the arcadal side of the sinus. Partial invasion of vascular endothelium by trophoblast establishes contact between the trophoblast and endometrial stroma.

## Methods

### Animals and tissue harvesting

Tissues were harvested from nine-banded armadillos (*Dasypus novemcinctus*) collected for the Yale Peabody Museum of Natural History (IACUC protocol #2014-10906). Animals were collected in Centerville, Texas, USA. The stages of pregnancy and the dates of collection are given in Table 1. The latest stage of pregnancy sampled in our specimens is the second stage of placental development, following Enders’ [12] criteria, i.e., after the vascular villi have formed but before the loss of cytotrophoblastic cell columns from the tips of villi.

**Table 1:** Animals used in this study

Animal ID	Stage of pregnancy	Crown-Rump length of fetuses	Date collected	Remarks
YPM0579	Non-pregnant (NP)	N/A	2014.05.17	
YPM0583	Post-implantation; before the establishment of vascular villi (early-gestation)	Microscopic embryo	2014.12.01	
YPM0584	After the establishment of vascular villi, and before the loss of cell columns from the tips of villi (mid-gestation)	22-25 mm	2015.01.06	
YPM0585	After the establishment of vascular villi, and before the loss of cell columns from the tips of villi (mid-gestation)	19-21 mm	2015.01.07	5 fetuses

Harvested tissues were fixed in 4 % paraformaldehyde for 12–24 h, and partially dehydrated by successive washes in 50 % and 70 % ethanol for an hour each. At this point, tissues were shipped from Centerville, TX to our lab on ice–packs and stored at −20 °C until further processing.

### Paraffin embedding and sectioning

Tissues were dehydrated with successive washes in ethanol, cleared in toluene, and embedded in paraffin blocks. Sections of thickness 2 μ or 5 μ were made on a microtome (Reichert-Jüng) and placed on poly-l-lysine coated glass slides.

### Haematoxylin and eosin staining (H&E)

Haematoxylin and eosin staining was performed following the standard procedure with 1–2 min in haematoxylin and 1 min in eosin.

### Masson’s trichrome staining

Protocol from [18] was followed. Slides were deparaffinized with xylene, brought to 70 % ethanol, and incubated overnight in zinc-formalin fixative at room temperature. Slides were dipped in deionized water (1 min), stained with Weigert’s iron-haematoxylin (3 min), and rinsed under running tap water (1 min). They were then stained with Biebrich Scarlet – Acid Fuchsin solution (4 min) and rinsed in acidified water to remove excess dye. The dye was further removed from collagen by immersing the slides in a solution of phosphomolybdic acid and phosphotungstic acid for about 10 min – this step was monitored under microscope to avoid excessive differentiation. Differentiation was followed by staining with 2 % Fast Green FCF (4 min), and two washes in acidified water (30 s each). Slides were dehydrated in ethanol, cleared in xylene, and coverslipped with Permount.

### Periodic Acid – Schiff (PAS) and Periodic Acid – Schiff – Diastase (PAS-D) staining

PAS and PAS-D staining was performed on serial sections to make the two comparable. Protocol from [18] was followed. Slides were deparaffinized with xylene and brought to water. For PAS-D staining, the slides were incubated with 1 mg/ml of α-amylase (i.e., diastase; Sigma-Aldrich) for 30 min at 37 °C, rinsed in tap water, and then with distilled water. For PAS staining, this step was performed with water instead of α -amylase solution. Slides were then oxidized for 30 min with 1 % solution of periodic acid, washed under running tap water for 3 min, and immersed in Schiff’s reagent for 20 min. Slides were washed under copiously running tap water for 10 min. Counterstaining was performed with Gill 2 haematoxylin for 1–2 min. Slides were dehydrated in ethanol, cleared in xylene, and coverslipped with Permount.

### Immunohistochemistry

Slides were heated at 60 °C in an incubator for 30 min, cooled at room temperature for 5 min, dewaxed in three changes of xylene (3 min each), and rehydrated by three changes in 100 % ethanol (3 min each) and by washing in running tap water for 5 min. Heat-mediated antigen retrieval was performed by heating the slides to 95 °C, and immersed in 10 mM Sodium Citrate buffer (pH 6.0) in a vegetable steamer. Slides, still immersed in the citrate buffer, were allowed to cool to below 30 °C. Slides were washed with PBS for 5 min and blocked in 0.1 % solution of BSA in PBS for 5 min. Endogenous peroxidases were suppressed by an incubation with Peroxidase Block (DAKO) for 30 min. Slides were incubated with an optimized dilution of primary antibody at 4 °C overnight in a humidified chamber. Secondary antibody was added to the slides after washing them with PBS for 5 min and blocking with 0.1 % BSA for 5 min. Incubation with secondary antibody was done at room temperature for an hour and slides were once again washed with PBS and 0.1 % BSA (5 min). If the secondary antibody was fluorescently labeled, slides were stained with DAPI (2 min; Sigma-Aldrich) and coverslipped using 50 % glycerol. If the secondary antibody was labeled with HRP, detection was performed with either DAB (DAKO) followed by counterstaining (Gill 2 haematoxylin) and coverslipping with 50 % glycerol, or TSA-cy3 (Perkin-Elmer) followed by counterstaining (DAPI) and coverslipping with 50 % glycerol. A list of antibodies used in this study is given in Table 2.

**Table 2:** Antibodies used in this study

Antibody product ID	Company	Antigen	Host species	Immunogen species	Label	Dilution used
ab9377	Abcam	Cytokeratin	Rabbit	Cow	none	1:50 (DAB); 1:200 (Alexa Fluor488)
ab6994	Abcam	vWF	Rabbit	Human	none	1:16000 (TSA-cy3)
sc6260	Santa Cruz	Vimentin	Mouse	Pig	none	1:200
ab28364	Abcam	CD31	Rabbit	Mouse	none	1:100
ab150073	Abcam	Rabbit-IgG	Donkey	Rabbit	Alexa Fluor 488	1:200
K4011	DAKO	Rabbit-IgG	Goat	Rabbit	polymer-HRP	Undiluted
A21422	Invitrogen	Mouse-IgG	Goat	Mouse	Alexa Fluor 555	1:200

## Results

### Vascular endothelium of the maternal blood sinuses

**Fig. 1:**
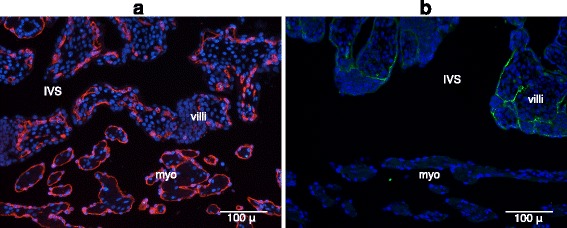
The interface between intervillous space and uterine tissue: myometrial side. **a** Bundles of muscle fibres in myometrium are lined by an endothelial layer (CD31 staining; red). **b** The layer around the bundles of muscle fibers in myometrium is not cytokeratin-positive (cytokeratin staining; green). IVS = Intervillous space, myo = Myometrium

In the placenta of our mid-gestation specimens, the maternal blood sinuses, in which the chorio-allantoic villi develop, have the endometrial arcade on the luminal side and the myometrium on the other. On the myometrial side of the intervillous space, the vascular endothelium is largely intact. Bundles of myometrial muscle fibers at the fetal-maternal interface are lined with an endothelial layer, i.e., endothelial marker-positive & cytokeratin-negative layer (Fig. 1a and b). Although no *decidua basalis* is present between the placental villi and myometrium (see Background), the intact endothelium precludes direct contact between villous and myometrial connective tissue.

**Fig. 2:**
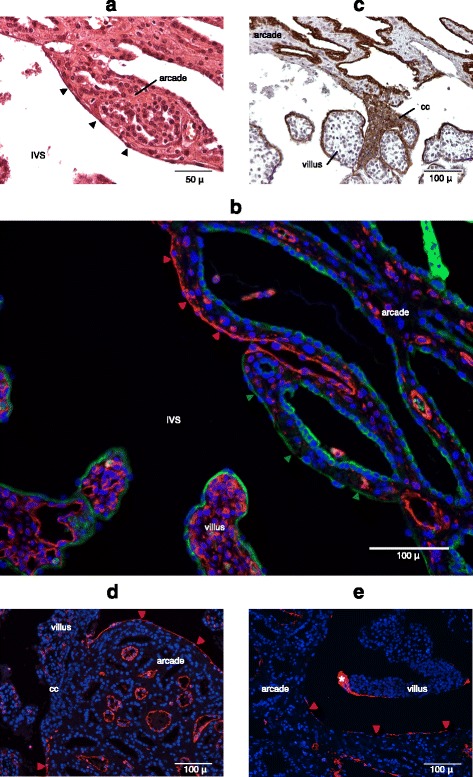
The interface between intervillous space and uterine tissue: endometrial arcade. **a** A thin and cellular layer, indicated by black arrowheads, delineates the boundary between intervillous space and endometrial stroma of the arcade. **b** The layer between intervillous space and endometrial stroma stains mutually exclusively with cytokeratin (green; indicated by green arrowheads) and vimentin (red; indicated by red arrowheads). **c** The cytotrophoblastic cell column is continuous with the cytokeratin-positive segment of the layer. **d** The layer stains discontinuously with CD31. CD31-negative portion of the layer is continuous with the cell columns. **e** The layer stains discontinuously with vWF. Asterisk shows perivillous fibrin-type fibrinoid. IVS = Intervillous space, cc = cell column of the cytotrophoblast

The endometrial arcade is separated from the intervillous space (i.e., maternal blood sinus) by a thin, cellular layer, visible in H&E stained histological preparations (Fig. 2a). This layer is thought to be the uninterrupted vascular endothelium of the maternal blood space. Surprisingly, segments of this layer stain positively for cytokeratin, a marker of epithelial and trophoblast cells. In order to confirm the identity of this layer, we performed an immuno-staining for markers of endothelial cells: CD31, von Willebrand factor (vWF), and vimentin (marks all mesenchymal cell). This layer stains positively for endothelial markers in discontinuous segments on the arcaded side of the fetal-maternal interface (Fig. 2b, d and e). Double staining with cytokeratin and vimentin shows that segments of this layer stain mutually exclusively with cytokeratin or vimentin (Fig. 2b), suggesting that segments of the vascular endothelium of the maternal blood sinuses are replaced by trophoblast cells, reminiscent of the replacement of endothelial cells in human spiral arteries by extravillous trophoblast cells. Indeed, the cytokeratin-positive segments of this layer are often seen in continuity with the villous cell columns of cytotrophoblast (Fig. 2c), perhaps depicting trophoblast in the process of replacing the endothelial lining or supplementing it to compensate for the stretching of the sinus. Similarly, Fig. 2d shows that the CD31-negative portion of the endometrial arcade is continuous with the cell columns. Double staining was only performed with vimentin and cytokeratin because our antibodies for CD31 and vWF are raised in the same species as the cytokeratin antibody. There is variation between animals in the degree of replacement of endothelium. Between the two mid-gestation specimens (see Methods), considerably more of the endothelium is replaced by trophoblast in YPM0584 than in YPM0585.

In sum, the vascular endothelium of the preformed blood sinuses remains intact on the myometrial side, but is partially interrupted and replaced by trophoblast cells on the arcadal side. It is the latter that can be termed as the junctional zone of the definitive armadillo placenta, where fetal and endometrial stromal tissue lie in direct apposition.

### Placental fibrinoid

**Fig. 3:**
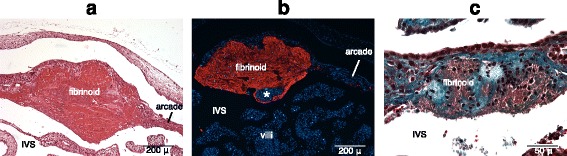
Placental fibrinoid. **a** H&E staining of a fibrinoid at the junction of arcade and intervillous space. **b** Fibrinoid is vWF-positive (vWF staining; red), therefore fibrin-type. It also looks continuous with the perivillous fibrinoid of a villus marked by an asterisk. **c** Fibrinoid stained with Masson’s trichrome method. Note that collagenous i.e., matrix-type and non-collagenous fibrinoid i.e., fibrin-type is intermingled. Also note the leukocytes present in the fibrinoid. IVS = Intervillous space

Placental fibrinoids are a-cellular and histologically glossy structures of either maternal or trophoblastic origin. We find that fibrinoids are present in our mid-gestation specimens. Fibrinoids were found regularly in the perivillous region (Fig. 2e), as well as in the endometrial arcade, on the side facing the intervillous space (Fig. 3a). Some of the fibrinoids in the arcade seem to be continuous with the perivillous fibrinoids of the villi that invade the endothelial lining (Fig. 3b). These fibrinoids are a combination of fibrin and matrix types. They stain positively with vWF, and contain numerous leukocytes trapped within them, implying that they are of the fibrin-type (Fig. 3b). Parts of these fibrinoids also contain collagenous material, which stains in shades of blue by Masson’s trichrome method, suggesting a partially matrix-type nature (Fig. 3c). The leukocytes present within the fibrinoids are primarily granulocytes based on their characteristic multi-lobed nuclei, and constitute the majority of the nucleated cells seen in these fibrinoids. It is unclear whether these fibrinoids are of maternal origin or trophoblastic.

Fibrinoids are not seen in H&E stained sections of the early-gestation stage of placentation studied, after implantation but before the establishment of vascular villi. Absence of fibrinoids at this stage was also confirmed by vWF immuno-staining and Masson’s trichrome staining (data not shown).

### Collagen

Endometrial extracellular matrix, especially collagens, undergoes substantial reorganization during pregnancy, which is necessary for accommodation of the growing fetus in the uterus, provision of a substrate for the placental structures and the immune cells at the fetal-maternal interface [19]. We performed Masson’s trichrome staining to document the dynamics of the collagen in armadillo endometrium during pregnancy.

**Fig. 4:**
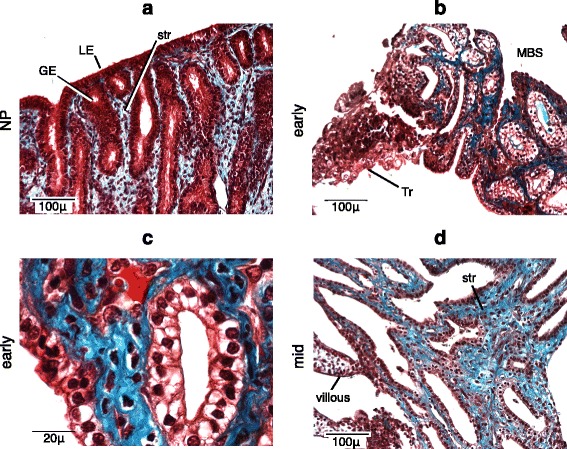
Collagen levels in the endometrial stroma increase during pregnancy. Masson’s trichrome staining. **a** Non-pregnant. **b** Early-gestation. **c** Early-gestation; higher magnification. Note the lack of cytoplasmic blue staining in some stromal cells. **d** Mid-gestation. GE = glandular epithelium, LE = luminal epithelium, str = endometrial stroma, MBS = maternal blood sinus, Tr = trophoblast

Collagen staining (blue) is weak in the non-pregnant endometrial stroma (Fig. 4a). The endometrium in the early-gestation stage, prior to the establishment of the vascular villi, is primarily occupied by numerous glands and preformed maternal blood spaces. The remainder, the endometrial stromal compartment, stains for collagen with remarkable intensity compared to the non-pregnant stage. The glandular epithelial cells in this stage are vacuolated (Fig. 4b), which may be a sign of secretory activity or glycogen storage (see below). At higher magnification, collagen seems to be present even in the cytoplasm of many endometrial stromal cells, suggesting that it is synthesized by these cells, consistent with their identification as a specialized fibroblast. We speculate that the stromal cells lacking the cytoplasmic collagen staining are immune cells (Fig. 4c), but immunohistochemical evidence for this interpretation could not be provided because we could not find antibodies that reliably stained leukocyte markers on armadillo tissue. Collagen staining decreases slightly in the stroma of endometrial arcades of mid-gestation specimens after the vascular villi have been established (Fig. 4d); nevertheless it remains stronger compared to the non-pregnant stage. Composition of the ECM with respect to specific collagen types was not determined due to the lack of armadillo-specific reagents, but it is clear that the overall levels of collagen increase considerably during pregnancy in the endometrial stromal compartment.

### Glycogen

We tested for the presence of glycogen-containing cells in the armadillo fetal-maternal interface by Periodic Acid- Schiff (PAS) staining in conjunction with amylase (diastase) digestion (PAS-D). Briefly, PAS stains glycogen, glycoproteins, and glycolipids. Comparison of sections stained with PAS, with and without prior amylase-digestion, is used to detect glycogen [18].

**Fig. 5:**
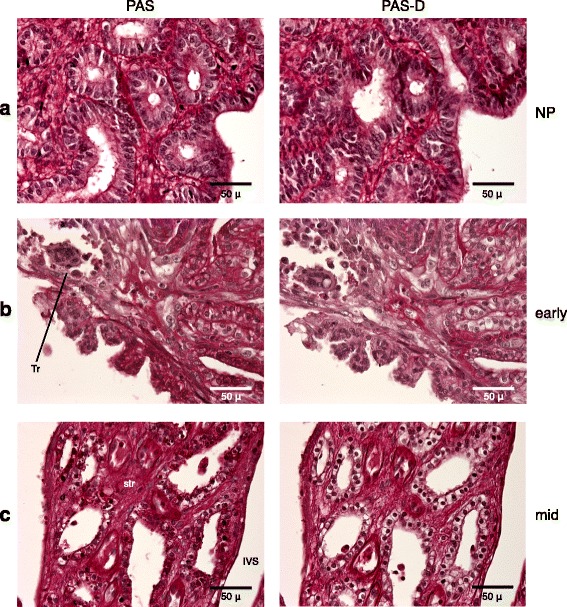
Glycogen detected by PAS/PAS-D staining. **a** Non-pregnant: no detectable glycogen in the endometrium **b** Early-gestation: intense glycogen staining in the luminal and glandular epithelia. **c** Mid-gestation; endometrial arcade: strong glycogen staining in the glandular epithelium and mild staining in the stroma. Tr = trophoblast, IVS = intervillous space

In the non-pregnant stage, the endometrial stroma is reactive to PAS, but it is largely amylase resistant, suggesting that there are no detectable glycogen-stores in the endometrial stromal cells at this stage. Epithelia are not reactive to PAS at this stage (Fig. 5a). In the early-gestation stage, endometrial stroma, as well as luminal and glandular epithelia, stain intensely with PAS reaction, but only the luminal and glandular epithelial staining is amylase-sensitive. Thus we conclude that at this stage the epithelia contain glycogen, while stromal cells do not. The invading trophoblast cells are devoid of PAS reactivity (Fig. 5b). In the mid-gestation stage, after the formation of vascular villi, a similar pattern is observed, with luminal and glandular epithelia being glycogen-positive. In this case, the stroma of the arcade is mildly amylase-sensitive, suggesting that there may be some glycogen-containing cells present, but the intensity is certainly much less than in the epithelium (Fig. 5c).

## Discussion

Many cell-types bearing glycogen stores are present at the fetal-maternal interface in rodents and human [20]. The precise function of glycogen stores in these cell-types is unclear, but they have been ascribed a nutritive function to the fetus [21], as well as have been proposed to be a necessary outcome of a hypoxic environment [22]. Here we showed that the epithelia of the endometrium are glycogen storing cells in the armadillo during pregnancy, consistent with earlier findings by Enders and colleagues [16]. It is possible that they provide the resources for the synthesis of mucopolysaccharides typically present on the luminal epithelia. Unlike in armadillo, in human [22] and rodent species [23], glycogen has also been described in endometrial stromal and decidual cells during pregnancy. It is not present in the armadillo endometrial stroma even in the early stage for which a decidual reaction has been described by Enders and colleagues [16]. We did not detect glycogen in the syncytial trophoblast of armadillo (data not shown), similar to the situation in human [22] and rodents, with the potential exception of rat [23].

Placental fibrinoids have been described in species with haemochorial placentation [13, 24–26]. There are two types of placental fibrinoid: fibrin-type and matrix-type. The former is primarily a blood clot, while the latter is rich in extracellular matrix proteins e.g., collagen, fibronectin, laminin [27]. Nelson and colleagues [26] have described fibrin-type fibrinoid in the term placenta of armadillo, masking the discontinuities within syncytial trophoblast layer surrounding the villi. We describe fibrinoids in our mid-gestation specimen of armadillo, even in specimens collected in January, two months before term. We also show that the fibrinoids are not limited to perivillous regions but are also seen in endometrial arcades. The remarkably high density of leukocytes present within the arcadal fibrinoids suggests a role in the regulation of immune reaction (see below).

A major enigma in reproductive biology is the question of how the maternal immune system tolerates the semi-allogenic fetus. Several mechanisms have been put forth to answer this question. In mouse these mechanisms include exclusion of cytotoxic T-cells from the fetal-maternal interface [28], regulatory T-cells [29], antigen presentation limited to the indirect pathway [30] or restriction of antigen-presentation altogether by the entrapment of dendritic cells in the endometrium [31]. Armadillo, with its haemochorial placenta, also faces this immunological challenge. Enders and Welsh [17] have argued that the peculiar way in which armadillo placenta develops—with little destruction of maternal tissue and little exposure of the endometrial stroma to the trophoblast—may help explain fetal-maternal tolerance. They propose that armadillo trophoblast is protected from the maternal immune system because most of the placental development takes place within a confined maternal blood space lined by an intact endothelium. There is minimal damage to the maternal tissue, and there is minimal contact between the fetal tissue and maternal connective tissue, only at the original site of invasion. Immune cells are typically inactive in circulation; an alloantigen provokes the immune system more potently when it is present interstitially, in the connective tissue, and in the context of an inflammatory reaction, rather than intravascularly, as in the armadillo placenta.

**Fig. 6:**
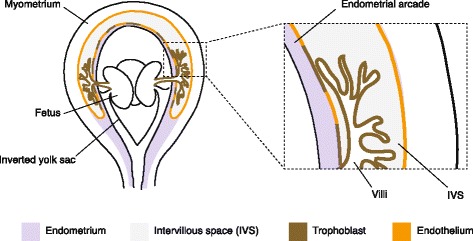
Illustration of the fetal-maternal interface in mid-gestation stage in longitudinal section. Note that endothelium of the intervillous space is partially replaced by trophoblast on the side of the endometrial arcade, while it is intact on the myometrial side. The illustration is adapted from [17]

Our results support the argument made by Enders and Welsh [17], with respect to the nature of fetal-maternal interface on the myometrial side. The endothelium of the maternal blood space is intact on the myometrial side, precluding a direct physical contact between the placental villi and myometrium, as envisaged by Enders and Welsh [17]. However, we show that the endothelium of the blood sinus on the side of endometrial arcade is partially replaced by trophoblast cells. This is reminiscent of the replacement of endothelial cells of maternal arteries by extravillous trophoblast in human [20]. In the case of armadillo the replacement of the endothelium may be initiated by the trophoblast cells of the villous cell columns. Armadillo trophoblast is thought to be invasive only during implantation [12, 13], but our observations suggest that it may be invasive even in the later stages of placental development, as long as the cell columns persist. The partial replacement of the endothelium by trophoblast brings fetal tissue in direct apposition with the endometrial stroma. In such an arrangement, the fetal tissue is not secluded from the maternal connective tissue anymore (Fig. 6), and therefore not completely protected from the possible inflammatory reaction by tissue-resident immune cells.

It should be noted that Rezende and colleagues [32] studied three species of armadillo (*Euphractus sexcinctus, Chaetophractus villosus and Chaetophractus vellerosus*), that did not include the one studied here, nine-banded armadillo (*Dasypus novemcinctus*), and concluded that the endothelium of maternal blood sinuses was intact. They validated this interpretation by vimentin staining in *Chaetophractus villosus*. In the paper, the authors have documented immunohistochemical evidence for presence of an intact endothelium only on the myometrial side. It is not possible to determine from that report whether the endothelium is intact on the side of endometrial arcade, which is where we found it interrupted in *Dasypus*.

The revised understanding of the armadillo fetal-maternal interface presented here calls for extension to the model of immune tolerance proposed by Enders and Welsh [17]. One possible mechanism for preventing an inflammatory response could be the placental fibrinoids. Fibrinoids have been hypothesized to suppress immune reaction by acting as an ‘immuno-absorbent sponge’ [24]. Although their role in armadillo placenta remains to be studied in detail, our study provides evidence for the presence of numerous leukocytes, potentially trapped, in the placental fibrinoids. Which additional means of immune tolerance armadillo employs is unclear at the moment, but the biology of interaction between armadillo mother and fetus should be explored further to answer that question. In particular, one would expect additional mechanisms ensuring limited inflammation and immune tolerance to be deployed in the endometrial arcade, the junctional zone of the armadillo placenta.

## Conclusion

We have documented the dynamics of collagen and glycogen at the fetal-maternal interface of nine-banded armadillo, and reported the presence of placental fibrinoids on the villi and in the endometrial arcade. We have also shown that the trophoblast of nine-banded armadillo invades the maternal tissue to a greater extent than was previously believed. It partially replaces the endothelial lining of the maternal blood sinuses and establishes a direct contact with endometrial stromal cells. This finding calls for further study of immunological tolerance and regulation of inflammation at the fetal-maternal interface in armadillo. Given its phylogenetic position, insights from armadillo, when combined with knowledge from other eutherian species, are critical for understanding the evolution of eutherian pregnancy.

## Abbreviations

BSA: Bovine serum albumin
cy3: Cyanine3
DAB: 3,3'-Diaminobenzidine
DAPI: 4',6-diamidino-2-phenylindole
PBS: Phosphate-buffered saline
TSA: Tyramide signal amplification
